# A Novel Approach to Measure Executive Functions in Students: An Evaluation of Two Child-Friendly Apps

**DOI:** 10.3389/fpsyg.2020.01702

**Published:** 2020-07-16

**Authors:** Valeska Berg, Shane L. Rogers, Mark McMahon, Michael Garrett, Dominic Manley

**Affiliations:** ^1^School of Arts and Humanities, Edith Cowan University, Perth, WA, Australia; ^2^Cinglevue International, Perth, WA, Australia

**Keywords:** executive functions, cognitive assessment, educational technology, students, classroom, cognitive functions, cognition, assessment

## Abstract

Interest in measurement of children’s executive functions has shown a major increase over the past two decades. The present study evaluates two new apps (EYT and eFun) for measuring executive functions in children. The results of this study show that children (aged 5–8) enjoy executive function assessment in the form of games on an iPad. However, only one executive function, EYT working memory, showed significant positive correlations with several types of grades (e.g., English and maths) in primary school students. New, self-assessed, child-friendly executive function measurement tools have the potential to provide future possibilities for teachers to integrate information on cognitive ability into student learning plans.

## Introduction

Over the past two decades, researchers from cognitive psychology, neuroscience and education have increasingly shown interest in measuring executive functions (EFs) in children (e.g. Miyake et al., 2000; Diamond, 2016; Zelazo et al., 2016). Recent attempts have been made to design child-friendly tools to measure EFs in children but a number of challenges persist (Zelazo, 2015; Howard and Melhuish, 2017; Józsa et al., 2017; Obradović et al., 2018; Holmboe et al., 2019; Willoughby et al., 2019). The current study presents the first step toward validating newly developed executive function tools. This study evaluates two novel child-friendly EF measurement tools specifically developed to assess EFs in the classroom. These are the Early Years Toolbox (EYT) developed by Howard and Melhuish (2017), and a new set of executive function assessment tasks called eFun (Berg et al., 2019). Both use technology-enhanced tasks rather than traditional paper-based assessments. A key consideration of our research was the children’s experience with these tasks as fun and enjoyable activities since it has been shown that enjoyment with a task can lead to greater task performance (Schukajlow and Krug, 2014).

To evaluate these tasks the following research questions were investigated: (a) Do children find the EF tasks enjoyable? (b) Does task performance across and within the two apps show positive associations? (c) Does the performance on the EF tasks positively associate with primary school grades?

The term “executive functions” is used to describe a set of interrelated cognitive processes that enable us to accomplish tasks and pursue goals by controlling cognition and behavior in a goal-directed manner (e.g., Gioia et al., 2001). The core triad of EFs are working memory, inhibition and cognitive flexibility (Miyake et al., 2000; Lehto et al., 2003; Diamond, 2013). Working memory (WM) is the ability to hold information in mind and manipulate it (Baddeley, 1992). WM is essential for remembering task requirements and organizing action plans (Diamond, 2013). Inhibition refers to the ability to deliberately stop or inhibit dominant or automatic behaviors and/or thoughts (e.g., Mäntylä et al., 2010). Inhibition is required to withhold inappropriate responses or thoughts and sustain attention to the task at hand. Cognitive flexibility is the ability to flexibly switch between and apply new and existing rules (Zelazo, 2015). This core EF is active when a situation or context requires the application or adaptation of a (new) set of rules. While these EFs are typically discussed as if they are relatively distinct, it is widely accepted in the literature that in everyday life activities EFs work together (Diamond, 2016; Zelazo et al., 2016).

When working in concert, these core executive functions allow a person to engage in the more complex cognitive processes necessary to solve everyday problems, such as planning and evaluating (Zelazo et al., 1997; Diamond, 2016). For example, when solving a task in school, rules or guidelines are kept in working memory and applied where needed. Additionally, distractors that interfere with a task are inhibited and flexible thinking is applied when switching between rules appropriate to the task at hand.

Indeed, executive functions build a foundation for learning and academic success (Posner and Rothbart, 2007; Zelazo et al., 2016). Executive functions predict math, English and science achievements in school (Bull and Scerif, 2001; St Clair-Thompson and Gathercole, 2006; Memisevic et al., 2018; Usai et al., 2018). Research has shown that teachers value their students’ EF capacities. For example, following classroom instructions while inhibiting distractions has been identified by teachers as a key element in a successful classroom (Lin et al., 2003). When not developed properly, EFs can result in learning difficulties. Executive function deficiencies can turn into severe behavioral issues including aggression, emotional disturbance, and criminality (Broidy et al., 2003; Denson et al., 2011). Therefore, it is not surprising that EFs are associated with overall quality of life (Moffitt et al., 2011).

Given the tremendous influence EFs have on success in school and life, EF levels need to be identified and addressed in a valid and reliable manner, with tools that are appropriate for use with children. The information gained from appropriate EF measurement tools can potentially provide teachers with a deeper understanding of students’ learning skills, which could, in turn, form the basis for individual learning plans in the future (Bierman et al., 2008; Flook et al., 2010; Diamond, 2012).

There are two main challenges for scholars who are interested in developing EF measurement tools for children. Firstly, EF tasks need to be child-friendly. The original executive function tasks designed for adults do not take into account the level of reading and writing ability a child possesses, or the limited attention span of a child. For example, one version of the traditional Go/No Go Task for adults requires the participant to respond to 600 stimuli, which can take up to 30 min to complete (Hackley et al., 1990). Other EF tasks, like the Stroop task (MacLeod, 1991) require the participants to read words. Therefore, researchers have recently tried to design new EF tasks to be more child-friendly by shortening task length and adapting the design and delivery method (e.g. Howard and Melhuish, 2017; Holmboe et al., 2019). Task instructions can be tailored for children by using child-friendly language to make it easier to understand and more engaging for children. Furthermore, tablets/computerized tasks allow for verbal (standardized) instructions given via headphones alongside visual interactive instructions on the screen. This eliminates instructor bias and limits the cognitive demands associated with social interactions. Nevertheless, although recent EF tasks attempt to be child-friendly, there is a lack of studies that evaluate how the child experiences the tasks.

If children enjoy playing the task it is more likely that they pay attention to the task, which can influence their performance on the task. In order to capture children’s attention and measure their full potential on the EF tasks, the eFun tasks were designed to be an enjoyable experience that the children like to play. Research has found that students that show enjoyment and interest in performance tasks score higher on the performance tasks (Schukajlow and Krug, 2014). Furthermore, task enjoyment has been found to be positively associated with attention and task persistence (Reeve, 1989; Engelmann and Pessoa, 2014), which leads to enhanced performance (Engelmann et al., 2009; Pessoa and Engelmann, 2010). Thus, for researchers designing new EF tasks, the challenge is not just the design of the task, but also the evaluation of children’s experience with the task. This lack of evidence to support assertions of child-friendliness is a common issue among tasks that have been recently designed for children (Cianchetti et al., 2007; Zelazo, 2015; De Greeff et al., 2016; Howard and Melhuish, 2017; Dawson and Guare, 2018; Holmboe et al., 2019; Willoughby et al., 2019).

The second challenge is that EF tasks need to be modified to suit *non-clinical* populations. EF tasks were originally developed to diagnose a small number of people with severe cognitive dysfunctions in a clinical context (e.g., Otto et al., 1991; Gold et al., 1997). However, identifying EF levels in *typically developing* children has recently attracted interest in research that aims to support children’s cognitive development (Howard and Melhuish, 2017; Holmboe et al., 2019). Therefore, there is a need to adjust the difficulty levels of the tasks to capture varying levels of EFs rather than only capturing severe executive dysfunction. Additionally, the initial clinical EF assessment tasks were originally designed to be conducted in decontextualized clinical settings that do not reflect how EFs operate in the everyday life of a child (Wallisch et al., 2018). This is problematic not only because the ecological validity of the tasks is low, but also because a child might not feel comfortable in an unfamiliar environment with one examiner assessing the child. This environment can induce stress or (test) anxiety in a child which may affect test performance (Shute et al., 2016).

A few recent attempts have been made to design child-friendly tools in order to measure EFs in children (Kado et al., 2012; Diamond and Wright, 2014; Zelazo, 2015; Howard and Melhuish, 2017; Józsa et al., 2017; Holmboe et al., 2019; Willoughby et al., 2019). Cognitive demands and assessment methods have been adjusted to make tasks more appropriate for children. For example, slight variations have been made to the stimuli and administration procedures and the length of EF tasks have been reduced to account for children’s limited attention span and (e.g., Howard and Melhuish, 2017).

To make EF tasks more appealing to children, several researchers have decided to use tablets instead of computers or physical tasks (Zelazo, 2015; Howard and Melhuish, 2017; Józsa et al., 2017; Holmboe et al., 2019; Willoughby et al., 2019). Using a tablet instead of a computer has several advantages (Falloon, 2013). Firstly, tablets require less attentional demands. The response location is on the tablet screen and not the computer keyboard, which means that the participants do not need to reorient their attention away from the computer screen to the keyboard. Reorienting attention can result in both additional time and effort, especially for children (Posner and Cohen, 1984; Hunt and Kingstone, 2003). Research confirms the benefits of this approach, showing that using a tablet instead of a computer is a more reliable measurement method for EF assessment in children, eliciting faster and better performances (Howard and Okely, 2015). Furthermore, tablets are mobile and can therefore be applied to different contexts, and they give the opportunity for self-administered testing, which eliminates instructor bias and costly instructor training. Finally, using an online-connected tool like a tablet enables fast data collection that can be uploaded and analyzed in a more efficient way than the traditional pen and paper recordings (Willoughby et al., 2019). Given the advantages of using a tablet, the current study employed this way of measurement to assess EFs in children.

### The Current Study, Evaluating Two New Child-Friendly Executive Function Measurement Tools

The current study is a beginning set of validity studies that presents the evaluation of two new EF measurement tools, the EYT (Howard and Melhuish, 2017) and eFun (Berg et al., 2019). The validation of educational and psychological test results is an ongoing process that requires multiple sources of evidence, with multiple samples (i.e., replication; Kane, 2013; American Educational Research Association et al., 2014). The present study represents an early stage in the validation assessment of eFun and EYT. Howard and Melhuish’s (2017) EYT consists of a group of tasks to measure executive functions in a child-friendly way with tasks that are short and easy to understand for younger children. The EYT consists of several publically available 2D EF apps. To measure the three core executive functions the toolbox has two working memory games, one inhibition task, and one cognitive flexibility task.

The EYT cognitive flexibility task “Card Sorting” is similar to the iPad version of the Dimensional Change Card Sort (DCCS) by Zelazo (2015). However, the EYT Card Sorting task requires less assistance than the DCCS because all instructions are given verbally through the app. In the visual-spatial working memory task called “Mr. Ant” children are asked to remember locations of dots on an ant. Dots are shown on the body of the ant and after a short delay children are asked to replicate the sequence of the previous shown dots. The second working memory task is called “Not This”. Children are presented with a number of different shapes that have cartoon faces on them. These vary in shape, size and color (e.g., small red triangle; large green circle). Children are given instructions to point to a shape that does not have a certain features (e.g., pointing to a shape that is not green/large/a circle). In lower levels children are asked to only hold one feature in mind, e.g., “find a shape that is not red”. In subsequent higher levels children are required to hold up to three features in mind, e.g., “find a shape that is not small, not blue and not a circle”. This task requires close monitoring by instructors, whereas the Mr. Ant WM task does not. The EYT inhibition task is adapted from the original Go/No-Go task (Donders, 1969). Children are either presented with a fish or a shark swimming from left to right on an iPad screen. Children are required to respond to the fish (“catch the fish”; go trial) by tapping the screen and to refrain from responding when the shark is displayed (“avoid catching sharks”; no-go trial). The majority (80%) of stimuli are fish (go trials), to generate a prepotent tendency to respond, while the tendency to respond has to be inhibited when the sharks are presented (the remaining 20%, which are no-go trials), for a detailed description see Howard and Okely (2015).

In the current study we also evaluated a newly developed self-assessed tablet EF measurement tool called “eFun” (Berg et al., 2019). All eFun tasks are based on established EF tasks which provided the foundation for the content of the eFun tasks. The newly built 3D eFun tasks are designed to measure executive functions in an engaging and child-friendly way in typically developing children. To make the eFun narration child-friendly, no numerical or letter knowledge is required. Furthermore, the tasks are brief and include dynamic elements to engage children’s attention. The eFun tasks were developed in collaboration with an educational software company using the Unity game engine, deployed on an Apple IOS tablet (iPad). A team of trained software engineers and researchers have collaborated on the eFun app to make it both engaging and based on principles from cognitive psychology.

**FIGURE 1:**
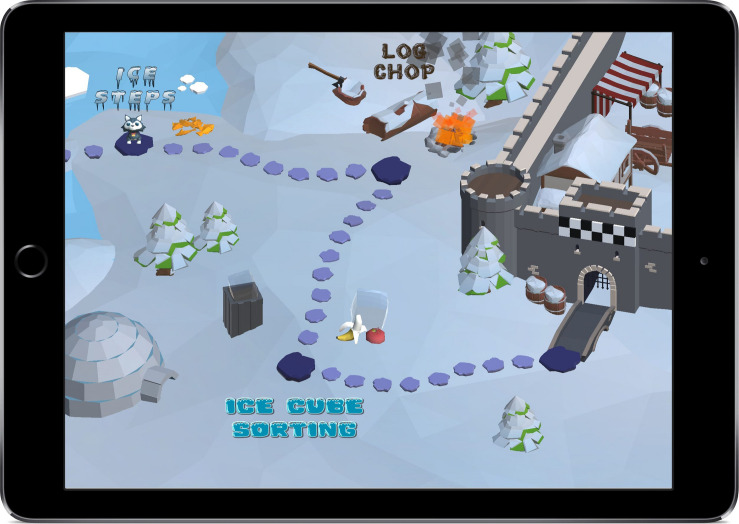
The eFun map screen showing the progressing through the three games. The Ice Steps game which assesses working memory, the Log Chop game which assesses inhibition, and the Ice Cube Sorting game which assesses cognitive flexibility.

In order to engage children, the eFun tasks offer advanced design elements with a variety of response mechanics (e.g., swiping, dragging and touching), along with high graphical fidelity and a 3D environment (see Figure 1). To eliminate instructor bias the eFun tasks are self-assessed through verbal instructions given via headphones. A narrator called “Owly” guides the child through the eFun winter world. Every task includes a story that outlines the overarching goal of the task. For example, in the inhibition game called “Log Chop” the child is asked to chop firewood to help keep the eFun villagers warm during an icy cold storm that hits the village. The logs/firewood are the “go” stimuli that need to be swiped in the inhibition task (for a detailed explanation of the tasks see the measures section). At the end of each task the children can see that they have achieved their goal via an end game screen (e.g., the villagers sit around the fire made of chopped wood).

Prior research suggests that providing a narrative has the potential to foster greater involvement with the task (Axelsson et al., 2016), especially if the participants are given a goal to work toward (Dickey, 2006; Lim et al., 2014). Furthermore, having a goal to work toward resembles real-life more closely and aligns with the definition of EFs being goal-directed processes (Gioia et al., 2001). In addition to that, including game elements in EF tasks has the potential to enhance intrinsic motivation. Dörrenbächer et al. (2014) found that adding game elements to computerized task-switching training enhanced intrinsic interest in the task. Thus, providing a narrative with clear goals in a game environment is thought to heighten involvement with tasks and increase ecological validity. However, this has not been previously researched in an executive function assessment context with children. Furthermore, unlike clinical tasks, the eFun games measure executive function in a classroom context that is familiar to the child and resembles real-life situations of the child more closely.

The current study contrasts children’s performance and experience of eFun with Howard and Melhuish’s (2017) EYT tasks. It was hypothesized that the inclusion of dynamic game elements and the overarching narrative of eFun would result in the participants reporting the eFun tasks as more engaging than the EYT tasks. Based on Howard and Melhuish (2017) findings, moderate to positive associations between matching tests *across* the two apps were expected (i.e., Inhibition_EYT_ and Inhibition_eFun_, CF_EYT_ and CF_eFun_, WM_EYT_, and WM_eFun_). Furthermore, the current study investigates the relationships between the core EFs *within* both the EYT and the eFun tasks. Based on the findings of Howard and Melhuish (2017) it was expected that the three EYT tasks will yield significant moderate inter-correlations among each other. Miyake et al. (2000) and Lehto et al. (2003) have shown that the core EFs are moderately correlated but clearly distinguishable in adults and 8–13 year old children, thus similar results were expected for the three eFun tasks in our sample (Miyake et al., 2000; Diamond, 2016). It was also hypothesized that the core executive functions measured with eFun and the EYT would predict school grades. The literature suggests that EFs positively associate with academic outcomes in school children (Bull and Scerif, 2001; St Clair-Thompson and Gathercole, 2006; Memisevic et al., 2018; Usai et al., 2018). In particular, working memory was expected to show the strongest positive correlation with academic outcomes (Bull and Scerif, 2001; St Clair-Thompson and Gathercole, 2006; Usai et al., 2018).

## Materials and Methods

### Participants

Two first and two second-year classes with a total of 81 students (54% girls; *M*_age_ = 6.98) from a primary school in Western Australia participated in the current study. Participants were recruited through the school with information letters that were handed out to parents by teachers (see procedure). Seventy-one participants were included in the analysis of the task ratings and 74 were included in the analysis of the task outcomes. Ten cases were excluded due to incomplete data in the EF task outcome data and seven cases were excluded due to incomplete data in the questionnaire data.

### Materials

Executive functions were measured with two test batteries on iPads. Three of the previously developed tasks in the EYT (Howard and Melhuish, 2017) and the newly developed eFun app (including all three tasks), were used in this study. The EYT can be downloaded from the iTunes app store. The EYT tasks have previously been reported to possess good reliability and validity (Howard and Melhuish, 2017). The iPads used in this study were provided by the school. Pen and paper was used to get feedback from the children on the EYT tasks (see the feedback questionnaire in the measures section below), whereas for the eFun tasks the feedback questionnaires were integrated into the app.

### Measures

#### EYT Tasks

These tasks are child-friendly executive function tasks that are part of the EYT developed by Howard and Melhuish (2017). For a more detailed description of the tasks beyond what is provided in this paper please see Howard and Melhuish (2017).

#### EYT Mr. Ant Task (Working Memory)

This task requires children to remember the spatial locations of dots on a cartoon ant. Working memory capacity is recorded as a point score (Morra, 1994). This is calculated by assigning one point for each consecutive level in which at least two of the three trials were answered correctly and 1/3 of a point for all correct trials thereafter.

#### EYT Go/No-Go Task (Inhibition)

This task requires children to respond to fish (go trial) by tapping the screen and to refrain from responding to sharks (no-go trial) that are swimming from the left to the right side of the screen. For the analyses, trials in which the response is faster than 300 milliseconds were removed, because Howard and Melhuish (2017) suggest that responses that are this fast are not likely to be in response to the target stimulus. This elimination is crucial for this task because it is not required to touch the stimuli on the screen to indicate a response; instead tapping the screen in any location is recorded as a response. Furthermore, if the participant does not respond to the majority of stimuli within one level (go accuracy below 20% and no-go accuracy exceeds 80%), or if the participant responds to all stimuli within one level (go accuracy exceeds 80% and no-go accuracy below 20%) then their data for that level is excluded (Howard and Melhuish, 2017).

#### EYT Card Sorting Task (Cognitive Flexibility)

In this task, children are asked to sort cards (e.g., red rabbits sitting on a raft, and blue boats) according to either color or shape into one of two locations: A castle with a flag displaying a blue rabbit, or a castle with a flag displaying a red boat. Scores are based on the number of correct sorts after the pre-switch phase.

#### eFun Tasks

These executive function tasks are based on established EF tasks (e.g. Donders, 1969; Milner and Taylor, 1972; Kado et al., 2012) and were newly developed as part of this research (Berg et al., 2019). Three discrete EF tasks were developed, based on the theoretical model by Diamond (2013) who proposes three core executive functions, and research by Miyake et al. (2000) and Lehto et al. (2003) showing that these three core EFs are separable.

#### eFun Ice Steps Task (Working Memory)

**FIGURE 2:**
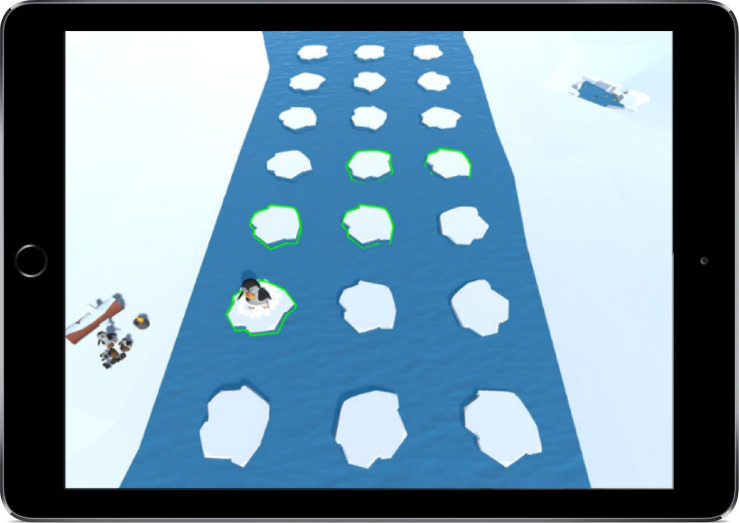
The eFun working memory game (Ice Steps) showing how Pongo the penguin crosses the river to feed the hungry chicks.

The Ice Steps task is based on the backward Corsi Block test (Milner and Taylor, 1972) and aims to measure working memory in children. The task starts with a brief introductory story explaining that Pongo the penguin needs to get fish for his chicks, which are on the opposite side of a river. The participant sees the hungry chicks crying for food. Next, the participant sees the penguin crossing a river on ice floats and getting fish for his chicks. The participant is asked to bring the penguin, with the collected fish, back to the chicks on the other side of the river. In order to get back to the other side of the river, the participant must remember the previously shown ice floats (organized in a grid, 3 × 8) in reversed order (see Figure 2). Following an explanation of how the game works, the child is asked to start the practice trial by tapping the series of ice floats the penguin previously used to cross the river in reversed order. The game starts with three ice floats to be remembered, increasing by one float to be remembered in each subsequent level. Each level consists of four trials with the same number of floats. In total there are four levels and the highest number of floats to be remembered is six. If the child gets three trials in one level wrong, the game is discontinued with a rewarding screen showing how the chicks get fed with the collected fish. Independent of the child’s performance, the task always ends with the rewarding screen.

Working memory is assessed with three measures that are based on scoring procedures of the backward Corsi Block test (Kessels et al., 2008). First, the longest sequence of ice floats that is correctly remembered backward (i.e., the span length; 3–6 floats) is recorded. Secondly, the number of correctly remembered trials (4 levels × 4 trials) is measured. Lastly, these two measures are combined with a product score, which is the span length multiplied by the number of correctly remembered trials.

#### eFun Log Chop Task (Inhibition)

**FIGURE 3:**
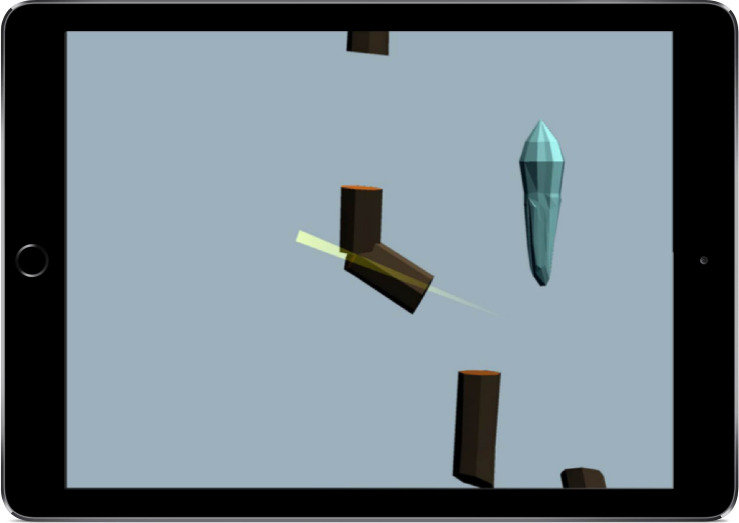
The eFun inhibition game (Log Chop) showing how the logs (go stimuli) get chopped.

The Log Chop task is based on the Go/No-Go tasks (Donders, 1969; Simpson and Riggs, 2006; Wiebe et al., 2012; Howard and Okely, 2015) and aims to measure inhibition in children. The task starts with a brief introductory story explaining that a storm has hit the eFun village and in order to keep the villagers warm the child needs to chop (swipe across) descending fire wood while avoiding reacting to descending icicles. After a practice phase, in which the child learns to respond to the wood but not the icicles, the actual game starts, see Figure 3. Independent of the child’s performance, the task always ends with a rewarding screen showing the characters around a fire made of the wood that the child had chopped.

The majority of stimuli in the log chop task are “go” stimuli (80% logs) to create a pre-potent tendency to respond. The task consists of three levels with increasing difficulty. Each level consists of 25 stimuli, 80% “go” stimuli (logs) and 20% “no-go” stimuli (icicles) and stimulus presentation (1.5 s) is held constant during all levels (based on Howard and Okely, 2015). The time between stimuli (the interstimulus interval, ISI) decreases from 1.5 s in level one to 1 s in level two and 0.5 s in level three. The decreasing ISIs act to speed up the task to increase difficulty. Additionally, no level starts with a “no-go” stimulus (icicle; Howard and Okely, 2015). The WM load is held constant throughout the game since the same rules apply in all levels and the rules are easy to remember (i.e., chop the logs, avoid the sharp icicles). Furthermore, the logs do not always appear in the same locations, thus responses cannot be anticipated (Aron, 2011; Diamond, 2013). Inhibition is indexed by the product of proportional go accuracy and proportional no-go accuracy (% go accuracy x % no-go accuracy; Howard and Melhuish, 2017). This score reflects the participants’ ability to withhold their response to the dominant pre-potent response.

#### eFun Ice Cube Sorting Task (Cognitive Flexibility)

**FIGURE 4:**
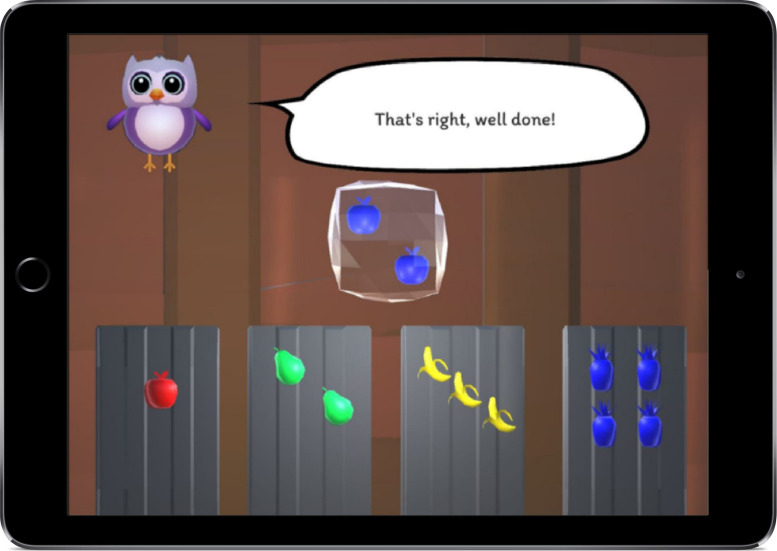
The eFun cognitive flexibility game showing the ice cube that needs to be sorted into one of the tubes. If the correct rule was “color” the cube would need to be sorted into the very right tube, for the rule “shape” it would need to be sorted into the very left tube and for the rule “number” it would need to be sorted into the second tube from the left.

The Ice Cube Sorting task is based on adapted card sorting tasks (Cianchetti et al., 2007; Kado et al., 2012) and aims to measure cognitive flexibility. The task starts with a brief introductory story explaining that Eski the husky wants to store food for the upcoming winter, since the husky might not be able to leave the house during the cold winter. After a brief practice phase, the child is asked to sort ice cubes containing fruit according to three sorting rules (color, shape, and number) into four tubes displaying four different fruit in four different colors ranging in quantity from one to four (e.g., one red apple, two green pears etc.), see Figure 4.

Cognitive flexibility is required because the switching demands in this task are high, while the working memory load and inhibition demand are kept at a constant level. The difficulty of the task increases as the task progresses by introducing more frequent rule switches. This frequency increases, from a new rule after every six correct sorts in level one, to a new rule after every three correct sorts in level two. In order to assist young participants, the rule changes are announced by the explanatory character and children are told that there are three different rules (color, shape, and number) in the beginning of the task. However, the participant does not know which rule needs to be applied next (color, shape or number). In order to find out which rule applies next, the participant needs to remember the previous rule (which cannot be applied again) and then test the two remaining rules. Based on feedback (“that’s right” or “that’s wrong”) the participant can find the correct sorting rule. Cognitive flexibility is measured with a perseveration error rate, which is the number of perseverative errors (PEs) divided by the number of rule changes. PEs occur if the child does not switch to a new rule after the rule switch has been announced, but instead continues to use the previous rule. A higher number of PEs reflects higher cognitive inflexibility (O’Donnell et al., 2017).

#### Feedback Questionnaire

After each task, the children were asked to fill out a brief questionnaire with seven questions assessing how enjoyable, fun, exciting, easy, hard, boring and frustrating they found the tasks. The questionnaire is based on the Intrinsic Motivation Inventory (IMI; Deci and Ryan, 2005), which is a multidimensional measurement device intended to assess participants’ subjective experience on a target activity. The IMI has been used in the context of intrinsic motivation and self-regulation assessment and includes questions assessing interest and enjoyment. For the purpose of this study with children, questions from the interest/enjoyment scale were adapted and a 4-point response scale was used: “no, not at all”, “a little bit”, “quite a bit” to “yes, a lot”. A very similar type of response scale has previously been shown to be clear and useful for studies involving young children (Rogers et al., 2016). For the EYT tasks, a pen and paper version of the questionnaire was filled out by the participants. The questions and answers were read out to the class to ensure that everyone understood them. For the eFun tasks, the questionnaire was integrated into the apps, therefore responses to the questions were given by tapping on a box underneath the question on an iPad screen. To accommodate non-readers, the questions were verbalized by the explanatory character (owl) and the questions and answers were verbally repeated if the child clicked on them.

#### School Grades

Grades ranging from “A” to “D” (for English, Math, HASS, Science, Design and Technology, and Digital Technology) were collected after the testing phase, during the mid-year break.

### Procedure

Before the study commenced, approval from both the Edith Cowan University Ethics Committee and the participating school was sought. The information and consent forms were sent to the school principal, the teachers and parents. The teachers distributed information and consent forms for the children to parents. The information letters that were given out outlined the procedure, possible risks, and purpose of the study. Additionally, the letters informed parents and teachers about a focus group session, in which children could express their opinion about the EF tasks that took place at the end of the study. In consultation with the teachers, appropriate times and dates for the data collection were determined. Before the first study commenced, the researchers were introduced to the students and teachers to familiarize the students with the people assisting the project. Unlike most existing tasks, all tasks were applied in an environment that is familiar to the child (the classroom). All students were tested at the same time in their classroom. Participants were asked to wear their own headphones that were stored at school. The first author (VB) was present throughout all testing sessions to ensure that the children were able to complete the tasks on their own. All instructions were presented verbally through the headphones in addition to being displayed in writing on the screen. Every participant went through two testing sessions: one to assess EFs with the EYT; and one to assess EFs with eFun. Both testing sessions were applied on the same day with a minimum break of two hours inbetween (based on the guidelines by Howard and Melhuish, 2017; and Straker et al., 2010). The individual testing sessions took no more than 25 min each (<50 min in total). The collected data was sent to a secure online database, ensuring confidentiality. The physical development guidelines for digital devices use by Straker et al. (2010) was taken into account when conducting this study.

### Research Design and Analysis

A non-experimental correlational design was chosen for this study. This means that no manipulation or selection into groups took place, but all variables of interest were assessed and analyzed. Analyses in SPSS were conducted to examine relationships between working memory, inhibition and cognitive flexibility within each test battery, as well as relationships between the eFun and EYT test batteries to assess convergent validity. Relationships between the variables were assessed using Pearson’s correlations. Moderate positive associations between matching tests across the batteries were anticipated (i.e., Inhibition_EYT_ and Inhibition_EFun_, CF_EYT_ and CF_EFun_, WM_EYT_ and WM_EFun_). Repeated measures analysis of variance (ANOVA) was conducted to examine if overall enjoyment of the tasks differed among the tasks.

Furthermore, after the initial measuring phase had ended, a brief focus group with the participants and the teacher took place to yield feedback on the tasks. The feedback informs the design of the eFun tasks for future research. The focus group was not conducted to assess the participants’ level of engagement with eFun or to explore the relationship between EFs and the measures within each task, but instead to gain insight into children’s opinions on the app design and to debrief children by addressing any issues they might have experienced during or after the testing phase.

## Results

### EF Task Ratings

**FIGURE 5:**
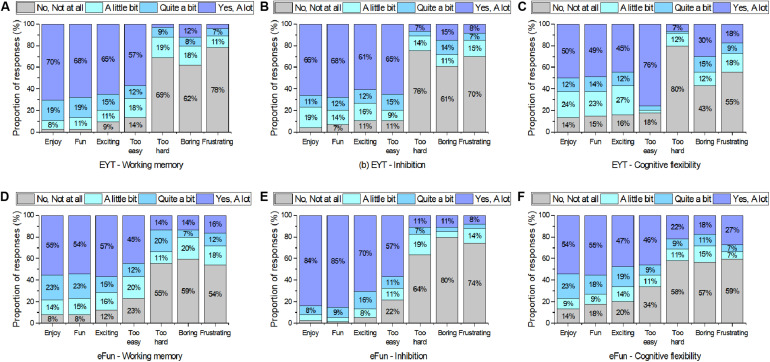
Student experience ratings for **(A)** EYT – Working memory, **(B)** EYT – Inhibition, **(C)** EYT – Cognitive flexibility, **(D)** eFun – Working memory, **(E)** eFun – Inhibition, and **(F)** eFun – Cognitive flexibility.

Answers to the EF questionnaires are presented in Figure 5. Overall, students experienced all EF apps as fun and enjoyable with, on average, over 50% of students reporting the tasks to be fun and enjoyable.

With regards to the eFun task ratings, the majority of students reported that they enjoyed the tasks and that they experienced them as fun (Figure 5). The eFun working memory and eFun inhibition tasks were reported to be exciting “a lot” by over 55% of students, whereas the eFun cognitive flexibility task was experienced as slightly less exciting (47% “a lot”) than the other two tasks. At the same time, over 40% of students reported that the eFun tasks were too easy “a lot” (45–57%). The inhibition task was perceived as the easiest eFun task, while also rated as the most enjoyable (84% “a lot”), the most fun (85% “a lot”), and the most exciting task (70% “a lot”) of all EF tasks in this study.

With regards to the EYT task ratings, over 60% of students reported the EYT WM and EYT inhibition task to be enjoyable and fun “a lot”. The EYT cognitive flexibility task received slightly lower enjoyment, fun, and excitement ratings than the other EF tasks. This task was perceived as the least exciting task (45% “a lot”) and the easiest task (76% “a lot”). Overall, the EYT tasks were perceived as too easy by the majority of children (57–76% “a lot”).

#### EF Task Ratings: ANOVA

In order to compare the enjoyment for each task, the ratings for the adjectives enjoy, fun, exciting, and boring (reverse scored) were combined into an enjoyment score. Inter-correlations among the ratings for the adjectives for each task were consistently moderate to high across all tasks. For example, the average inter-correlation among the eFun CF task adjectives (fun, exciting, boring, enjoy) was 0.71. Therefore, for each task a composite overall enjoyment score was created.

A one-way repeated measures analysis of variance (ANOVA) was applied to compare the student’s ratings on the EF tasks from the two test batteries eFun and EYT. The ANOVA results show that the participants enjoyed playing some tasks over others *F*(5,365) = 13.32, *p* < 0.001, *n_*p*_^2^* = 0.15). The follow up pairwise comparison showed that the eFun inhibition task (*M* = 3.64, *SD* = 0.55) was significantly more enjoyed than the EYT inhibition task (*M* = 3.30, *SD* = 0.86), *t*(73) = 3.13, *p* = 0.003, *d* = 0.047. There was no significant difference (*p*-values were greater than 0.05) between the enjoyment rating of the EYT WM task (*M* = 3.43, *SD* = 0.79) and the eFun WM task (*M* = 3.23, *SD* = 0.84) and no significant difference was found between the EYT cognitive flexibility task (*M* = 2.87, *SD* = 1.10) and eFun cognitive flexibility task (*M* = 3.10, *SD* = 1.02).

### Distributions of Performance Scores Among the EF Tasks

**FIGURE 6:**
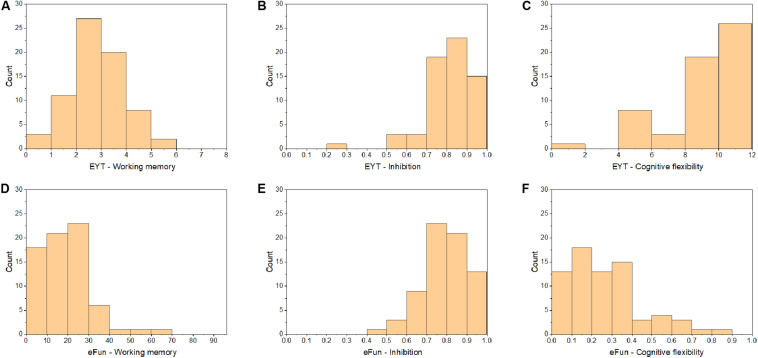
Frequency histograms of student performance on **(A)** EYT – Working memory, **(B)** EYT – Inhibition, **(C)** EYT – Cognitive flexibility, **(D)** eFun Working memory, **(E)** eFun Inhibition, and **(F)** eFun Cognitive flexibility.

The distribution of students’ task performance on each task is shown in Figure 6. The frequency histograms show that there were distributional issues with all tasks except the EYT WM. The EYT WM shows a normal distribution whereas the other tasks are skewed toward the higher or lower end of the scoring range. Looking over the distributions (see Figure 6) it is apparent that overall some tasks were too easy (EYT inhibition, EYT cognitive flexibility, eFun inhibition) while others were too difficult (eFun working memory, eFun cognitive flexibility). Note that higher scores (perseveration error rate) on the eFun cognitive flexibility task indicate worse performance.

### Correlations Between Grades and EF Tasks

Overall, there was one task, the EYT WM, that showed consistent moderate significant correlations with grades. Scores on the EYT WM task were significantly correlated with all subjects (significant correlations ranging between 0.32 and 0.50), see Table 1. In addition, scores on the eFun WM task were significantly related to Math grades. However, this correlation (*r* = 0.25, *p* < 0.05) is lower than the correlation between the EYT WM task and grades (*r* = 0.34, *p* < 0.05).

**TABLE 1:** Correlations Between Grades and EF Tasks.

	English	Math	HASS	Science	Design and tech.	Digital tech.
eFun WM	0.20	0.25*	0.20	0.18	0.23	0.16
eFun Inhib.	0.07	0.05	0.02	0.07	0.06	0.05
eFun CF	–0.02	–0.04	–0.12	–0.14	–0.08	0.01
EYT WM	0.38*	0.34*	0.50*	0.35*	0.47*	0.32*
EYT Inhib.	0.06	0.03	0.05	0.08	–0.07	–0.07
EYT CF	0.06	0.02	0.18	0.18	0.13	0.12

EYT = Early Years Toolbox, *p < 0.05.

#### Correlations Within and Between EYT and eFun

Contrary to expectations, when looking at correlations among working memory, inhibition, and cognitive flexibility *within* each of the apps no significant correlations were observed. There was a single exception where the EYT cognitive flexibility task and EYT inhibition task showed a low correlation of *r* (69) = 0.24, *p* = 0.049.

With regards to convergent validity *between* the two task batteries, the only significant correlation was found between the eFun and the EYT cognitive flexibility tasks, *r* (69) = -0.24, *p* = 0.041. The negative correlation can be explained by the different scoring procedures for the tasks. Higher scores (perseveration error rate) on the eFun cognitive flexibility task (Ice Cube Sorting task) indicate worse performance whereas higher scores (number of correct sorts after the preswitch phase) on the EYT cognitive flexibility task (Card Sorting task) indicate better performance.

## Discussion

This study evaluated two newly developed executive function (EF) assessment tools (i.e., EYT and eFun) on an iPad for children in a classroom environment. Building on the knowledge of previous research on child-friendly EF measurement tools (Howard and Okely, 2015; Howard and Melhuish, 2017), this study extends the literature on executive functions assessment in children by targeting (a) children’s opinion on the task (b) a new child-friendly design, and (c) a new assessment environment. Results showed that children enjoyed playing both the EYT and eFun tasks in a game-like fashion on an iPad in the classroom. Children’s evaluation of the assessment tool has rarely been taken into account when measuring EFs in children in previous research.

This article presents an early stage in the validation argument of the eFun and EYT test results. The validation arguments are based on the “Standards” for educational and psychological testing (American Educational Research Association et al., 2014). The “Standards” provide guidelines for assessing the validity of interpretations of test scores. According to the “Standards”, one source of validity evidence is evidence based on the test content. The task content for this study was developed in accordance with EF theory and existing EF testing formats. Furthermore, this study tested the task content for appropriateness for the given age group of the sample by asking students for their opinions on the tasks (student questionnaires and focus groups). We were also able to determine from the task performance results that some tasks were too easy and others too hard (ceiling effects). This feedback will be used to further improve the tasks in future iterations. It also became apparent that some children with disabilities such as vision or hearing impairments were not able to undertake the tasks. Thus future research should investigate task modifications to include children with disabilities in the assessment process.

Another source of evidence is based on the response process. This is an aspect of validity that needs to be further investigated in future research. Cognitive processes that are activated during EF testing with EYT and eFun could for example be assessed by neuroimaging (Collette et al., 2006; Cristofori et al., 2019) or “talk aloud protocols”. The latter method qualitatively explores how the children are conducting the tasks by recording their thought processes while they are playing the tasks. This approach would support the assessment of strategy use and help to better understand the thought process of children while playing the tasks (Schrier, 2017; Bratiotis et al., 2019).

Evidence for validity can also be assessed with the analysis of the internal structure of a test. For this study interrelationships between variables were assessed and are in line with theory stating that the core EF constructs are distinct (Miyake et al., 2000). Future research might reassess these relationships with a larger sample and/or within a more diverse target group. Relationships with other external variables also offer a source of evidence for validity. In this study, EF performance was expected to positively associate with academic outcomes, therefore we analyzed correlations between EF scores and grades. We plan on conducting future research to retest these relationship with larger and more diverse samples. Convergent evidence was assessed by investigating the relationship between EFs measured by two different types of assessment (i.e., EYT and eFun). As these types of assessments undergo further refinement, we plan on retesting convergence between these tasks and similar tasks. Lastly, consequences of testing can support the validity argument. A consequence of this study will include teachers’ access to the EF test results of their students, which can support the development of targeted learning plans for students. Future research is needed to investigate how well these tasks can be utilized by teachers to better understand and educate their students.

The EF eFun tasks developed for this research aim to measure students’ full potential and it has been shown that students that show enjoyment and interest for performance tasks score higher on the performance tasks (Schukajlow and Krug, 2014). Thus, enjoyment with the task should not be neglected when measuring children’s task performance. Exploring the relationship between enjoyment and (the related concept of) task-motivation is an important research area for future EF research, however, this is outside the scope of the current study. Executive function tasks and feedback questionnaires could for example be complemented with a task motivation questionnaire (please see Eklöf (2006) for a detailed explanation). Furthermore, the results of this study show that children enjoyed playing the tasks, which can be seen as a positive consequence of the task (Kane, 2013), however, the consequences of the results for students and teachers carry more weight in the validity argument than the enjoyment.

Furthermore, we acknowledge that while established original EF tasks have provided a foundation for the newer tasks that were used in the present study, the tasks in this study differ to the extent that the validity of these tasks cannot be argued based on this basic similarity. The similarity lies in the underlying cognitive mechanism such as remembering something in reversed order (to measure WM) but task response methods and design have been adapted, e.g., from tapping physical blocks to tapping ice floats on an iPad. It will take multiple studies to accumulate an evidence base to provide enough evidence to have full confidence in the more “gamified” tasks that are emerging in the literature (such as EYT and eFun) as alternatives to the more traditional tasks.

### Executive Function Task Ratings

The majority of children enjoyed playing the EYT tasks in addition to over half the children reporting that the EYT tasks are too easy. A possible explanation for the large number of students reporting that the EYT tasks were too easy is that these tasks were designed for children aged 3—6, whereas the age of the students in the current sample was marginally above this age (5–8 years). The EYT cognitive flexibility task (Card Sorting Task) was rated as the easiest and the least exciting and fun task out of all EF tasks in this study. In a focus group that was conducted after the study to inform the design of EF tasks in the future, students explained that the EYT cognitive flexibility task instructions felt too repetitive. The verbal instructions (“now we play the color/shape game”) were repeated before every trial. Thus, a future consideration when designing EF tasks for children is to give visual feedback/instructions instead of repetitive verbal instructions.

The majority of children also reported that they enjoyed the eFun tasks and thought they were fun. The highest enjoyment was reported for the eFun inhibition task (Log Chop). It is important to also note that of the eFun tasks collectively, the inhibition task was perceived as the easiest. Yet, at the same time, it was rated as the most enjoyable, fun and exciting task out of all EF tasks applied in this study. This suggests that in the current study children perceive less challenging tasks as more enjoyable. In contrast, literature on challenge and enjoyment typically reports that participants find challenging tasks more enjoyable (e.g., Shernoff et al., 2014). However, when separating voluntary and non-voluntary tasks, research suggests that non-voluntary tasks are enjoyed most when they are of low challenge (Koestner et al., 1987). This further demonstrates one of the challenges of designing enjoyable, yet valid measurement tools for children. If the tasks are too easy, outcome scores result in ceiling effects, despite children enjoying them more. Thus, future research needs to find an appropriate level of challenge that enhances rather than inhibits enjoyment in order to create valid and child-friendly measurement tools.

The eFun inhibition task was enjoyed significantly more than the EYT inhibition task. A possible explanation for this finding is that the design of the eFun task is different to the EYT task in terms of the level and nature of stimuli and interaction. In the eFun task, stimuli (logs and icicles) are moving vertically from the top to the bottom of the screen in varying stimuli locations (i.e., left, middle, and right), whereas the EYT has stimuli (fish and shark) moving horizontally from the left to the right only in the middle of the screen. The response mechanics also differ in that the eFun task requires swiping of the stimuli and the EYT requires tapping the screen. The screen in the EYT task can be tapped anywhere to indicate a response, whereas the eFun task requires the children to “chop” the logs with their fingers. Thus, response locations cannot be anticipated in the eFun task, increasing the difficulty and dynamic nature of the task. Furthermore, the speed of the presentation of stimuli in the eFun increases as the levels get higher, which makes it a highly dynamic game.

### Executive Functions and Grades

As expected, the results showed a link between working memory (measured with the EYT app) and academic outcomes in primary school children. This is in line with prior research showing that working memory is related to school achievement (van der Sluis et al., 2007; Bull et al., 2008; Sesma et al., 2009; Latzman et al., 2010; Usai et al., 2018). In particular, math (in grade 1-3; Monette et al., 2011; Usai et al., 2018) and English (in 11 and 12 year olds; St Clair-Thompson and Gathercole, 2006) have previously been found to be linked to working memory in children. A recent study investigated associations between the core EFs measured at kindergarten entry and its long term effects on academic achievement in grade 3 (Nguyen and Duncan, 2019). Similar to the results of the current study, Nguyen and Duncan (2019) found that working memory had the strongest associations with math and reading achievement, with math showing the strongest association, whereas inhibition and cognitive flexibility were found to have weaker links with reading and math achievement. Other research supports these findings (Greenfader, 2019), underlying the importance of working memory for academic achievement.

However, it should be noted that the EYT working memory task might measure a slightly different construct that is closer to short-term memory than to working memory. Items need to simply be rehearsed only and not repeated in reverse order in the EYT task; rehearsing items without manipulation has been argued to measure short-term memory rather than working memory (Baddeley, 2012). Higher variability and a normal distribution of the outcome scores for the EYT memory task might explain why this task was found to be related to school grades.

The other EF constructs, inhibition and cognitive flexibility, did not show a relationship with grades in the current study. There are two possible explanations for why no other significant links between EFs and academic achievement were found. Firstly, the participants enjoyed playing the tasks and scored relatively well on the tasks, which is reflected in the data, with relatively low variability across task performances (see Figure 6 in results section). A lack of variability in the outcome data makes it more difficult to differentiate high performers from low performers, which in turn, limits the potential to find significant results. This is a general measurement issue in research, especially with children (Jacobs and Paris, 1987; Reynolds and Mason, 2009). One potential reason for the lack of variability in the EF tasks is because the EF tasks were based on original EF tasks that were designed to identify executive dysfunction in clinical populations. For example, the Wisconsin Card Sorting Test (WCST) was initially used to identify people with various types of brain dysfunction (e.g., Milner and Petrides, 1984) and to assess neuropsychological dysfunction in school-aged children with developmental psychopathologies (Pennington and Ozonoff, 1996). Thus, in order to assess more subtle levels of varying EF abilities in a typically developing population, we argue that the difficulty levels of the tasks used in the present study could benefit from revision.

A second possible explanation for the low correlations between EF tasks and grades is the age group of the children in this study. In primary school children executive functions are still developing and therefore are more difficult to assess at this younger age. For example, inhibition emerges and undergoes rapid growth in early childhood, particularly between the ages of three to six (Band et al., 2000; Carver et al., 2001; Garon et al., 2008; Wiebe et al., 2012), and continues to mature into early adulthood. Considering the age group of the current sample (5–8 years), inhibitory skills have started to emerge but might not be fully developed yet. Furthermore, children have lower attention spans and therefore are more likely to disengage with the task, which can result in data that is less reliable than data collected with older participants. This is reflected in the research literature, which often reports positive relationships between EF task performance and academic achievement in *older* children (11–16 years; 9–15 years; Sesma et al., 2009; Latzman et al., 2010). This highlights the need for more research with appropriate tools on EFs in relation to academic achievement at the beginning of primary school.

### Correlations Between EYT and eFun

Convergent validity between the two task batteries was found to be low, as low or non-significant correlations were found between matching EF tasks across eFun and EYT. The only significant correlation was between the cognitive flexibility tasks, and it must be noted this was only a small correlation. One reason for the non-significant correlations for working memory and inhibition between the two task batteries could be that the EYT is designed for a younger age group. The EYT tasks are designed for children aged 3–6 (Howard and Melhuish, 2017), and eFun is designed for children aged 5–9 (Berg et al., 2019). Additionally, the eFun test battery has higher difficulty levels than EYT. Another important difference between the tasks is the design. As discussed earlier, the response methods and mechanics differ in the inhibition tasks. Additionally, the constructs that were measured might have differed, especially in the working memory tasks. The eFun WM task exposes a more complex memory construct, since it asks participants to keep stimuli in mind and indicate them in reversed order. On the other hand, the EYT measures a more simple (or short term) memory construct by asking the participants to simply repeat the order of stimuli without having to reverse it. Similarly, the eFun inhibition game is more complex because the stimuli locations vary and the speed increases, whereas the EYT inhibition game does not change stimuli location or the pace by which the stimuli are appearing. In contrast, the cognitive flexibility tasks are both measuring a very complex construct, which might explain the statistically significant low correlation found between these two tasks.

### Task Inter-Correlations

In the current study, there were small or non-significant correlations among EF tasks which is consistent with EF theory by Miyake et al. (2000) that suggests the three core EFs (working memory, inhibition, and cognitive flexibility) are separable constructs. Results showed no significant associations between the EF constructs, with the exception of EYT Inhibition and the EYT cognitive flexibility task, which showed a low significant correlation. Therefore, based on our data, the EF constructs WM and inhibition are distinguishable in the current sample of primary students.

Considering the supporting theories that postulate that executive functions consist of three core constructs (e.g., Miyake et al., 2000; Diamond, 2013), using three tasks to measure executive functions is deemed appropriate in this study. However, the findings of the current study do not support research that shows that the three core executive functions are moderately correlated (Miyake et al., 2000; Lehto et al., 2003). The findings need to be interpreted with respect to the population that the theories are based on. Miyake et al. (2000) conducted their research with undergraduate students and Lehto et al. (2003) investigated EFs in typically developing teenagers aged 15 and 16 years. Future research is needed to better understand at what age executive functioning becomes developed to the extent that there are consistent inter-correlations among assessment tasks.

### Limitations

The main limitation of this study was the sample. First, the number of participating children was relatively low. Second, only students in year one and two from one private primary school were included. Additionally, students with severe sensory impairments, such as vision or hearing impairments were not able to take part in the study. Thus the results may not be representative of the typical range of primary students in Australia. The nature of the sample may have also acted to reduce the variability of the data in the present study. Therefore, future research is needed with a larger sample that is more diverse.

An issue in the present study was ceiling effects within our EF performance data. Ceiling effects are a consistent issue within the literature where scholars aim to measure EFs in children, especially when measuring inhibition (Holmboe et al., 2019; Willoughby et al., 2019). For example, Willoughby et al. (2019) found floor and ceiling effects on several inhibition tasks with children (Go/No-Go, Silly Sound Stroop tasks, and Spatial Conflict Arrows). The authors also found low correlations among the executive function tasks in their study and mention that limited task variation in the Go/No-Go task was a problem (for more information please see Willoughby et al., 2019). Similarly, Petersen et al. (2016) and Holmboe et al. (2019) mention variability issues with regards to inhibition tasks. Petersen et al. (2016) explain that ceiling and floor effects are associated with lower variability in the measured construct, which increases Type II error and reduces power to detect associations with other variables. Another reason for floor and ceiling effects can be a small number of trials. Generally, tasks for children are kept short to suit their limited attention span, however, having a small number of trials increases the risk of low variability and ceiling effects in children (Holmboe et al., 2019). Thus, researchers interested in measuring inhibition with children need to be cautious with their selection of trials and outcome variables in order to avoid low variability associated with floor and ceiling effects.

### Implications

The new EF tablet-based measurement tools have useful implications for teachers. The EF apps are an enjoyable activity that can easily be implemented in the classroom schedule, as they do not require any assistance. The outcome data can be uploaded to an online cloud storage, which has potential to give teachers quick access to data about their students’ skills. Knowing about students’ EF levels can facilitate the development of targeted learning plans. Thus, the purpose of the online EF assessment tool eFun is not to diagnose students but to help teachers to better understand their students’ learning profile. For example, during the data collection period of our research one student scored very high on the WM task and passing that information onto the teacher helped the teacher gain a greater understanding of the student’s particular cognitive profile. Additionally, pointing out the student’s strengths to the student has the potential to increase that student’s confidence levels.

Nevertheless, the eFun EF apps are at an early stage of development and still need to be refined to fit primary students’ skill levels and to increase variability of the outcome scores. More specifically, this means that the tasks need to be challenging, but enjoyable (with simple and clearly communicated rules) for primary school children. We are planning to adjust the eFun app accordingly and to extend the app by the inclusion of an additional game measuring problem-solving. The problem-solving task is hypothesized to involve all three core executive functions (Zelazo et al., 1997; Diamond, 2013).

## Conclusion

The EF tasks that are typically used in the research literature are based on neuropsychological tests that were originally used to diagnose executive dysfunction. The EF tasks used in the present study were modified to be appropriate for use with typically developing children. We examined a newly developed set of EF tasks (eFun; Berg et al., 2019). As expected, children self-reported experiencing all the tasks as fun and enjoyable. Nevertheless, it is an ongoing process to redesign the original tasks to suit participants with a more typical cognitive development profile. This study has contributed to this movement in that we have identified areas that need improvement and have been able to measure children’s perspectives on EF tasks for the first time. The challenge of future research is to address the issues identified by refining EF measurement tasks that can maintain valid results, while being easy to use, particularly for primary school-aged children for whom the development of EFs can impact greatly on the early stages of their learning journey.

## Data Availability Statement

All datasets presented in this study are included in the article/Supplementary Material.

## Ethics Statement

The studies involving human participants were reviewed and approved by Edith Cowan Ethics Committee. Written informed consent to participate in this study was provided by the participants’ legal guardian/next of kin.

## Author Contributions

VB is the lead author who conducted the research, developed the measurement tool, and wrote and edited the manuscript. SR edited the manuscript and contributed to the research process and measurement tool development. MM and MG edited the manuscript and contributed to the measurement tool development. DM is the lead developer of the measurement tool and helped with the acquisition of data. All authors contributed to the article and approved the submitted version.

## Conflict of Interest

VB is studying under an industry-based Ph.D. scholarship, which is financially supported jointly by Edith Cowan University and Cinglevue International. Resources that were utilized in the development of the eFun app were provided by Cinglevue International. Cinglevue International intends to incorporate this into their Virtuoso platform and offer it to customers on a commercial basis. The remaining authors declare that the research was conducted in the absence of any commercial or financial relationships that could be construed as a potential conflict of interest.
